# Phase conjugation of twisted Gaussian Schell model beams in stimulated down-conversion

**DOI:** 10.1515/nanoph-2021-0502

**Published:** 2021-11-03

**Authors:** Gustavo H. dos Santos, Andre G. de Oliveira, Nara Rubiano da Silva, Gustavo Cañas, Esteban S. Gómez, Stuti Joshi, Yaseera Ismail, Paulo H. Souto Ribeiro, Stephen Patrick Walborn

**Affiliations:** Departamento de Física, Universidade Federal de Santa Catarina, Florianóplis, SC CEP 88040-900, Brazil; Departamento de Física,Universidad del Bío-Bío, Collao 1202, 5-C Concepción, Chile; Departamento de Física, Universidad de Concepción, 160-C Concepción, Chile; ANID – Millennium Science Initiative Program – Millennium Institute for Research in Optics, Universidad de Concepción, 160-C Concepción, Chile; Optics and Photonics Centre, Indian Institute of Technology Delhi, Hauz Khas, New Delhi 110016, India; Quantum Research Group, School of Chemistry and Physics, University of KwaZulu-Natal, Durban 4001, South Africa

**Keywords:** phase conjugation, stimulated down-conversion, twist phase, twisted Gaussian Schell-model

## Abstract

Stimulated parametric down-conversion is a nonlinear optical process that can be used for phase conjugation and frequency conversion of an optical field. A precise description of the outgoing stimulated field has been developed for the case where the input pump and seed fields are coherent. However, partially coherent beams can have interesting and important characteristics that are absent in coherent beams. One example is the twist phase, a novel optical phase that can appear in partially coherent Gaussian beams and gives rise to a nonzero orbital angular momentum. Here, we consider stimulated down-conversion for partially coherent input fields. As a case study, we use twisted Gaussian Schell-Model beams as the seed and pump beams in stimulated parametric down-conversion. It is shown both theoretically and experimentally that the stimulated idler beam can be written as a twisted Gaussian Schell-Model beam, where the beam parameters are determined entirely by the seed and pump. When the pump beam is coherent, the twist phase of the idler is the conjugate of that of the seed. These results could be useful for the correction of wavefront distortion such as in atmospheric turbulence in optical communication channels, and synthesis of partially coherent beams.

## 1 Introduction

Parametric nonlinear processes play a central role in classical and quantum optics, as they can give rise to amplification, modulation and frequency conversion of optical fields. Typically, the input fields are assumed to be spatially and temporally coherent. However, many modern light sources display only partial transverse coherence. Moreover, partially coherent beams can have interesting and useful characteristics. One example is the Twisted Gaussian Schell-model (TGSM) beam [1, 2], which carry a so-called twist phase, and could be useful in communication through turbulence [3, 4] and imaging [5].

The role of partial transverse coherence in nonlinear optics has been studied for the case of spontaneous parametric down-conversion [6–17]. A related process, known as stimulated parametric down-conversion (StimPDC), occurs when a pump and a seed beam interact in a medium with a nonzero second-order susceptibility [18]. They produce a third field, known as the stimulated or idler field, which can be the phase conjugate of the seed [19–22]. Phase conjugation can be used to correct wavefront distortion [23], with applications in laser construction [24, 25], high-resolution imaging [26], and communications [27–29]. StimPDC has also been used to produce quantum states of light [30–33]. Using an appropriate source, StimPDC has been used for phase conjugation and frequency conversion of vector beams [22, 34], [35], [36], and as a tool for probing the spontaneous regime with an increased signal [37].

Even though StimPDC has been recently addressed in several different contexts, including studies using structured light, the role of partial transverse coherence in the process has not yet been addressed. Here, we consider partially coherent StimPDC, with the aim of elucidating the relationship between the transverse degree of coherence as well as the twist phase of the pump, signal and idler beams. This knowledge will help to enhance the applications of TGSM beams. Section 2 develops a general description of the output idler field in terms of monochromatic but partially coherent pump and seed beams. We then apply this to the case in which both input fields are described by the TGSM [1, 2]. In Section 3 we present results of a StimPDC experiment using a TGSM seed beam with a coherent pump beam, observing that the idler beam is the phase conjugate of the seed.

## 2 Partially coherent StimPDC

Let us first recall StimPDC using spatially coherent beams in a single, thin nonlinear crystal. For a sufficiently strong seed beam, and considering that the crystal length *ℓ*_*z*_ is much smaller than the Rayleigh length of the pump beam *z*_R_, it was shown in Refs. [19, 35] that the output idler field is described by

(1) $$\phi \left(q_{i}\right)=\iint dq_{s}P\left(q_{s}+q_{i}\right)S^{*}\left(q_{s}\right),$$

where P(q) is the angular spectrum of the pump beam, and $S^{*}\left(q_{s}\right)$ is the angular spectrum of the seed field. The vectors of the form **q** = (*q*_*x*_, *q*_*y*_) are the transverse wave vectors of the seed (*s*) and idler (*i*) fields. Taking the Fourier transform, the outgoing idler field profile is

(2) $$\Phi \left(r\right)=P\left(r\right)S^{*}\left(r\right),$$

where *P* and *S* are the field profiles of the pump and seed beams, respectively. The above expressions assume beams with perfect transverse coherence.

A simple and intuitive approach to StimPDC with partially coherent beams is to consider that every partially coherent beam can be decomposed as a convex sum of coherent beams. Thus, for partially coherent pump and seed beams, this results in a statistical mixture of coherent StimPDC processes, each with coherent pump beam and seed profiles given by *P*_*j*_(**r**) and $S_{k}^{*}\left(r\right)$ , respectively. The output idler field is no longer described by a coherent field as in (2), but rather by the cross-spectral density (CSD) function

(3) $$W_{i}\left(r,r^{\prime }\right)=\underset{j,k}{\sum }\lambda_{j}\beta_{k}P_{j}^{*}\left(r\right)S_{k}^{*}\left(r\right)P_{j}\left(r^{\prime }\right)S_{k}\left(r^{\prime }\right),$$

where the weighting coefficients *λ*_*j*_ and *β*_*k*_ are nonnegative and each sum to unity. One can recognize

(4) $$W_{p}\left(r,r^{\prime }\right)=\sum \lambda_{j}P_{j}^{*}\left(r\right)P_{j}\left(r^{\prime }\right),$$

as a coherent mode decomposition [38] of the CSD function of a partially coherent pump beam. A similar expression can be written for the seed beam. Moreover, we can recognize the CSD function of the idler field (3) as the product of the CSD function of the pump beam with the complex conjugate of the CSD function of the seed beam:

(5) $$W_{i}\left(r,r^{\prime }\right)=W_{p}\left(r,r^{\prime }\right)W_{s}^{*}\left(r,r^{\prime }\right).$$

We note that this expression is quite general, considering only that the pump and the seed be monochromatic paraxial fields, incident on a thin nonlinear crystal. In the next section, we apply this result to the special case of TGSM beams.

### 2.1 StimPDC with twisted Schell-model beams

There are several types of partially coherent beams. Perhaps the most well-known is the Gaussian Schell-model (GSM) [38], whose intensity and degree-of-coherence profile follow Gaussian distributions. A more general class are the TGSM beams [1, 2, 39, 40], which include a novel twist-phase *μ*. The TGSM beams reduce to GSM beams when *μ* = 0. These are Gaussian beams, which at the beam waist are described by the CSD function

(6) $$\begin{array}{ll} & T\left(r,r^{\prime },\mu \right)\\ & \;=A{\text{e}}^{-\frac{r^{2}+r^{\prime 2}}{4w^{2}}}{\text{e}}^{-\frac{{\left(r-r^{\prime }\right)}^{2}}{2\delta^{2}}}{\text{e}}^{-\text{i}k\frac{\left(r^{2}-r^{\prime 2}\right)}{2R}}{\text{e}}^{-\text{i}k\mu \left(xy^{\prime }-yx^{\prime }\right)}, \end{array}$$

where *w* is the beam waist, *δ* is the transverse coherence length, and *R* is the radius of curvature. In the near-field, the intensity distribution has variance $\sigma_{\text{nf}}^{2}=w^{2}$ . The positivity constraint on the CSD function determines that the twist phase *μ* satisfies *k*|*μ*| ≤ 1/*δ* ^2^ [1, 2].

Considering both the seed and pump as TGSM beams, and using Eq. (6) in Eq. (5), the idler field can be written as

(7) $$\begin{array}{ll} & W_{i}\left(r,r^{\prime }\right)\\ & \;=A{\text{e}}^{-\frac{r^{2}+r^{\prime 2}}{4w_{i}^{2}}}{\text{e}}^{-\frac{{\left(r-r^{\prime }\right)}^{2}}{2\delta_{i}^{2}}}{\text{e}}^{-\text{i}k_{i}\frac{{\left(r-r^{\prime }\right)}^{2}}{2R_{i}}}{\text{e}}^{-\text{i}k_{i}\mu_{i}\left(xy^{\prime }-yx^{\prime }\right)}, \end{array}$$

suggesting that the output idler field can be described by a TGSM beam with beam waist

(8) $$w_{i}^{2}=\frac{w_{s}^{2}w_{p}^{2}}{w_{s}^{2}+w_{p}^{2}},$$

transverse coherence length

(9) $$\delta_{i}^{2}=\frac{\delta_{s}^{2}\delta_{p}^{2}}{\delta_{s}^{2}+\delta_{p}^{2}},$$

phase curvature

(10) $$\frac{k_{i}}{R_{i}}=\frac{k_{p}}{R_{p}}-\frac{k_{s}}{R_{s}},$$

and twist phase

(11) $$k_{i}\mu_{i}=k_{p}\mu_{p}-k_{s}\mu_{s},$$

where *i*, *s*, *p* stand for idler, signal (seed) and pump, respectively. Taking the absolute value of Eq. (11) and using (9), we note that the positivity constraint on the twist phase is satisfied, namely:

(12) $$k_{i}|\mu_{i}|\leq k_{p}|\mu_{p}|+k_{s}|\mu_{s}|\leq 1/\delta_{p}^{2}+1/\delta_{s}^{2}=1/\delta_{i}^{2},$$

showing that the output idler field can indeed be understood as a bona fide TGSM beam. The above equations show that the parameters of the TGSM seed and pump beams can be used to engineer TGSM idler beams.

Equation (9) gives the coherence length of the idler field as a function of the coherence lengths of the seed and pump beams, from which we see that the transverse coherence length of the idler field is always less than or equal to that of the least coherent beam *δ*_*i*_ ≤ min(*δ*_*s*_, *δ*_*p*_). For example, if *δ*_*s*_ = *δ*_*p*_, then the coherence length of the idler is $\delta_{i}=\delta_{s}/\sqrt{2}$ . However, since coherence length is always considered with respect to the beam waist, it is more illustrative to consider the ratio

(13) $$\frac{\delta_{i}}{w_{i}}=\sqrt{\frac{\delta_{s}^{2}\delta_{p}^{2}}{\delta_{s}^{2}+\delta_{p}^{2}}\frac{w_{s}^{2}+w_{p}^{2}}{w_{s}^{2}w_{p}^{2}}}.$$

When *ω*_*p*_ = *ω*_*s*_ and *δ*_*p*_ = *δ*_*s*_, the ratio of coherence length and beam waist of the idler field remain the same as that of the pump or signal (seed) photon (*δ*_*i*_/*w*_*i*_ = *δ*_*s*_/*w*_*s*_ = *δ*_*p*_/*w*_*p*_). However, the beam waist and coherence length of the idler field are each related to those of the pump/seed by a factor of $1/\sqrt{2}$ .

When the pump beam has zero twist phase (*μ*_*p*_ = 0), then from Eq. (11) the idler twist phase *μ*_*i*_ = −(*k*_*s*_/*k*_*i*_)*μ*_*s*_, so that the twist phase of signal and idler are equal in amplitude when the fields are degenerate. The idler twist phase is thus the opposite of the seed, but the two beams are not necessarily complex conjugates of one another, due to the other beam parameters. Specifying further that the pump beam described by a coherent plane wave ( $\delta_{p}^{2}\gg \delta_{s}^{2}$ , *μ*_*p*_ = 0, $w_{p}^{2}\gg w_{s}^{2}$ , *k*_*s*_/*R*_*s*_ ≫ *k* *_p_*/*R* *_p_*), we have *w*_*i*_ ≈ *w*_*s*_, *δ*_*i*_ ≈ *δ*_*s*_ and *k*_*i*_ *μ*_*i*_ = −*k*_*s*_ *μ*_*s*_, so that, aside from a possible change in wavelength, the output idler field is

(14) $$W_{i}\left(r,r^{\prime }\right)=T_{s}\left(r,r^{\prime },-\mu_{s}\right)=T_{s}^{*}\left(r,r^{\prime },\mu_{s}\right),$$

showing that the outgoing idler beam is the phase conjugate of the incoming seed beam. In the next section, we confirm these theoretical results in a StimPDC experiment.

The term phase conjugation is very often related to the idea of a light beam reflected by a phase conjugation mirror, which produces a copy of the input beam propagating backwards in time. In the context of StimPDC, it is also possible to produce an idler beam that is a copy of the seed beam evolving backwards in time. However, it is not a reflection, and this occurs when the pump can be approximated by a plane wave. A more general way of understanding the phase conjugation that occurs in StimPDC is by envisioning the idler beam as the field resulting from the scattering of the seed beam in a modulator described by the pump beam, which propagates backwards in time. The effective modulation provided by the pump can change the wavelength, amplitude and phase of the idler as compared to the input seed beam.

## 3 Experiment

**Figure 1:**
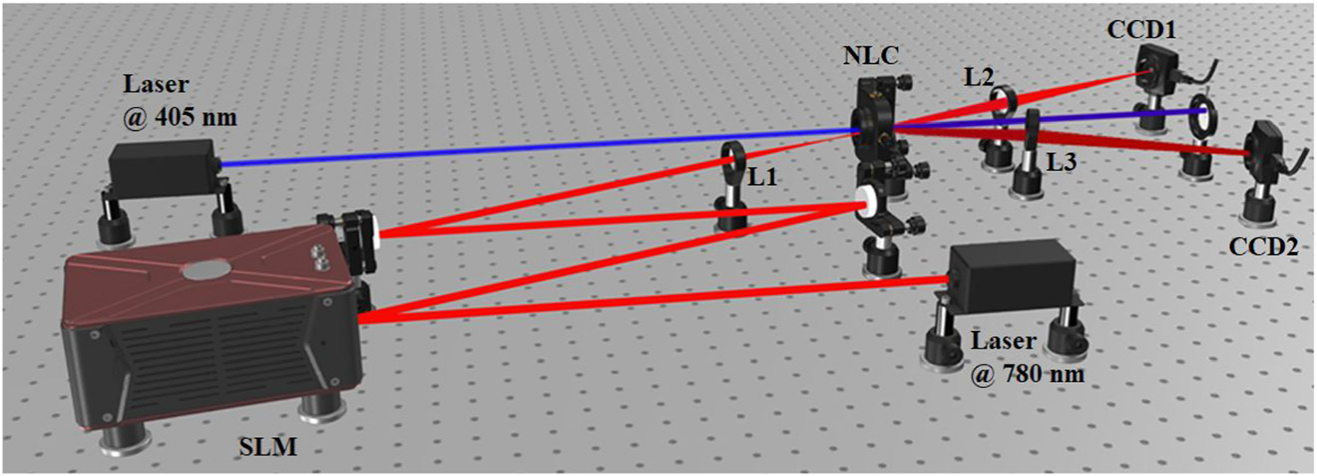
Experimental setup. A pump beam is incident on a nonlinear crystal producing parametric down-conversion. An auxiliary laser is used as a seed to stimulate the parametric emission aligned with the signal beam. It is prepared as a TGSM beam using a spatial light modulator (SLM), where a ( ∼20s ) movie of 300 phase-randomized phase/amplitude masks is used to produce the partially coherent seed field. Both seed and idler beams after the crystal are measured with a CCD camera.

Figure 1 shows a sketch of the experimental setup. It is a typical StimPDC experiment [34, 36], where a 2 mm long BBO (Beta-Barium-Borate) nonlinear crystal is pumped by a diode laser oscillating at 405 nm. The phase matching conditions for the crystal are type I, so that the vertically polarized pump gives rise to horizontally polarized signal and idler at 780 and 840 nm, respectively. In order to achieve stimulated emission in the idler mode, a seed laser beam (780 nm) is input into the seed mode.

The key difference from previous StimPDC experiments is the use of a partially coherent seed beam, which is prepared as a TGSM beam. It is produced by sending the initially coherent seed laser onto a spatial light modulator (SLM) that is broadcasting a sequence of phase-randomized images, following the methods demonstrated in Refs. [41–43]. To achieve partial coherence, we use a video composed of 300 images played at 15 frames per second, giving a total sampling time of 20 s. Each image is given by a blazed grating pattern to maximize transmission into the first diffraction order. Upon the blazed grating, we imprint a phase profile corresponding to

(15) $$\Phi_{l}\left(r\right)=\overset{N}{\underset{n=1}{\sum }}\sqrt{p\left(v_{n}\right)}K\left(r,v_{n}\right){\text{e}}^{\text{i}\varphi_{l,n}},$$

with

(16) $$\begin{array}{ll} K\left(r,v\right) & =\exp\left[-\frac{w^{2}}{2aw^{2}+1}\right.\\ & \;\left.\times {\left(\frac{r}{2w^{2}}+ar-av\right)}^{2}-ik\mu \left(xv_{y}-yv_{x}\right)\right] \end{array}$$

and weight function

(17) $$p\left(v\right)=\exp\left(-\frac{av^{2}}{\left(2aw^{2}+1\right)}\right),$$

and the parameter $a=\frac{1}{\delta^{2}}\left(1+\sqrt{1-k^{2}\mu^{2}\delta^{2}}\right)$ . Thus, each image is composed of *N* = 529 Gaussian functions displaced by the vector **v**_*n*_ with cartesian components sampled uniformly in the range $\left[-2\sqrt{\left(2w^{2}+1/a\right)},2\sqrt{\left(2w^{2}+1/a\right)}\right]$ . The phases *φ*_*l*,*n*_ are chosen randomly between 0 and 2*π*, so that the Gaussian functions can be considered mutually incoherent [41–43] when averaged over the 300 images in each video. Upon averaging, the output field is given by ∑_*l*_Φ_*l*_(**r**_1_)Φ_*l*_(**r**_2_) ≈ *T*(**r**_1_, **r**_2_). After preparing the seed beam in the SLM, lens L1 is used to project the image of the SLM surface onto the crystal plane with a magnification factor of 0.56. Lenses L2 and L3 are used to perform the optical Fourier transform of signal and idler fields at the crystal plane to the detection plane, at which CCD1 and CCD2 are used to record images of the Fourier-transformed seed and idler fields with an integration time of 1 s. A total of 20 CCD images are recorded and the final intensity distributions are obtained by summing over the entire set of images. For the purposes of this experiment, the pump beam is prepared as a collimated Gaussian mode with width 0.5 mm. The pump beam is coherent, with transverse coherence length *δ*_*p*_ ≫ *δ*_*s*_. All beams were prepared with a large radius of phase curvature *R* ≫ *z*_R_ corresponding to an approximately flat wave front.

In what follows, we classify TGSM beams according to the normalized twist phase *τ* = *μkδ* ^2^, such that −1 ≤ *τ* ≤ 1. We prepare the seed beam with normalized twist phases *τ*_*s*_ = 0, ±1. For each twist phase, we perform measurements for seed and idler beams while varying the transverse coherence length *δ*_*s*_ of the seed. In a first set of measurements, we measure the far-field variance $\sigma_{\text{ff}}^{2}$ . In the second, we measure the visibility of interference fringes produced in a double-slit experiment.

## 4 Results

### 4.1 Coherence transfer

**Figure 2:**
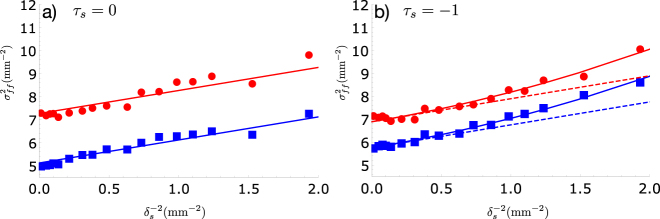
Transverse variance in the far-field as a function of the inverse square of the transverse coherence length ( $1/\delta_{s}^{2}$ ) for the seed (red circles) and idler (blue squares) beams. Error bars are smaller than the symbols. In (a) *τ*_*s*_ = 0 and (b) *τ*_*s*_ = −1. The variance of the idler beam mimics the seed, showing that the transverse coherence of the idler follows that of the seed beam. The distinct *y*-intercepts are due to slightly different phase curvatures. See main text for additional details.

The first set of measurements was carried out with the goal of studying the transverse coherence properties of the stimulated idler beam, as a function of the properties of the seed beam. With the coherent and collimated pump beam, and the seed beam prepared as a TGSM beam, from Section 2.1 we expect *δ*_*i*_ ≈ *δ*_*s*_ and *μ*_*i*_ = −*k*_*s*_ *μ*_*s*_/*k*_*i*_ ≈ −*μ*_*s*_ since the fields are nearly degenerate (*λ*_*i*_/*λ*_*s*_ ∼ 1.08). A common method for studying the coherence properties of TGSM beams is through the divergence of the beam [44]. Here, we use the CCD camera to measure the variance of the intensity distribution in the far-field $\sigma_{\text{ff}}^{2}$ of both the seed and idler, as a function of the transverse coherence length *δ* of the seed beam. The expected far-field variance from (6) is

(18) $$\sigma_{\text{ff}}^{2}=\left(\frac{1}{4w^{2}}+\frac{k^{2}w^{2}}{R^{2}}\right)+\frac{1}{\delta^{2}}+\frac{\tau^{2}w^{2}}{\delta^{4}},$$

showing that the beam diverges faster for nonzero twist phase. Figure 2 shows plots of the far-field variance $\sigma_{\text{ff}}^{2}$ of both the seed and idler beams versus the inverse square coherence length of the seed beam $1/\delta_{s}^{2}$ . Here, we scale both the coherence length and beam width to account for the magnification factor of the imaging system from the SLM to the image plane at the crystal. In Figure 2a we see that the far-field variance of the seed beam increases linearly with the parameter $1/\delta_{s}^{2}$ as expected for normalized twist phase *τ* = 0. The solid lines have slope equal to one, following (18). The idler beam behaves nearly identically to the seed beam, indicating that the transverse degree of coherence of the idler follows that of the seed. Similar results are observed when the seed is a TGSM beam with *τ*_*s*_ = − 1, shown in Figure 2b. However, in this case the solid lines correspond to plots of $a+\delta_{s}^{-2}+b\delta_{s}^{-4}$ , with *a* and *b* free parameters. For *τ*_*s*_ = −1 we obtain *b* = 0.29 ± 0.04 mm^2^ for the seed and *b* = 0.27 ± 0.02 mm^2^ for the idler beam, showing that the idler beam diverges nearly identically to the seed. These values are consistent with the theoretical value of *b* = *τ* ^2^ *w* ^2^ ≈ 0.3 mm^2^. For comparison, the dashed lines have slope equal to one and *y*-intercept given by *a*, showing the increase in divergence due to the nonzero twist phase. The different *y*-intercepts between seed and idler in all plots, which correspond to the term in parentheses in (18), can be attributed principally to a slightly different phase curvature of the beams. This is due to the fact that the curvature of the idler beam depends upon both the seed and the pump, following (10). The similarity between the far-field variances of the seed and idler beams partially confirm (14).

**Figure 3:**
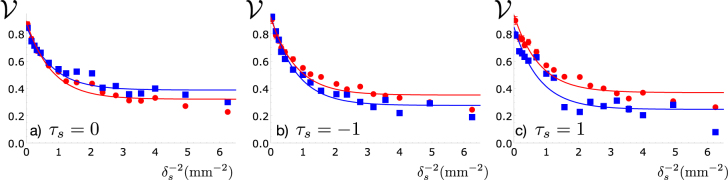
Visibility in Young double-slit interference as a function of $1/\delta_{s}^{2}$ for both the seed and the idler beam. The normalized twist phases are (a) *τ*_*s*_ = 0, (b) *τ*_*s*_ = −1 and (c) *τ*_*s*_ = 1. The visibility serves as an indicator of transverse coherence. Thus, these results show that the transverse coherence of the idler beam follows the seed. Error bars are smaller than the symbols.

As an additional test of the coherence properties, in a second set of measurements we have analysed the visibility of the interference fringes in a double-slit experiment as a function of the transverse coherence length. The double-slit aperture was imprinted over the TGSM images on the SLM, and this profile was imaged onto the crystal plane. The CCD camera was again placed in the far-field of the double-slit, as shown in Figure 1. The resulting interference fringes were accumulated over 20 s (all 300 TGSM frames). The visibility V was calculated by curve fitting the fringe patterns to a typical interference function of the form $I\left(y\right)=A\;\exp\left(-y^{2}/B^{2}\right)\left[1+V\;\cos\left(\left(y-y_{0}\right)/C\right)\right]$ . Figure 3 shows the visibility as a function of $1/{\delta_{s}}^{2}$ for the seed and idler beams. In both cases, the visibility decays exponentially with the inverse square coherence length. The maximum value for large *δ*_*s*_ is around 0.8, due to the finite transverse coherence of the seed laser beam incident on the SLM. For small *δ*_*s*_, the minimum visibility is around 0.3, due to a small background of coherent laser light that is produced by the SLM. To take into account these imperfections, we fit the data using the function $V=V_{0}+D\;\exp\left(-2d^{2}/\delta_{s}^{2}\right)$ , with 2*d* = 0.8 mm the center-to-center distance of two slits. These results demonstrate that the transverse coherence of the idler follows that of the signal, in this particular case where the pump beam is a fully coherent Gaussian beam.

### 4.2 Transfer and conjugation of twist phase

The results presented in Figure 2 confirm the presence of twist phase in the seed and idler beams given in Eq. (14). What remains is to observe the phase conjugation relation between the TGSM seed and idler beams. To do so, we exploit the fact that the 2D double-slit interference pattern is rotated by an amount proportional to the twist phase, with far-field intensity pattern give by:

(19) $$I\left(r\right)\approx \left[1+{\text{e}}^{-\frac{2d^{2}}{\delta^{2}}}\;\cos\left\{2dk\left(\frac{y}{f}-\mu x\right)\right\}\right],$$

where *f* = 400 mm is the focal length of the far-field optical system (lenses L2 and L3), and 2*d* is the distance between the centers of the slits. Thus, the central interference maximum lies along *y* = (*fμ*)*x*. We can thus use the interference patterns to estimate not only the amplitude but also the sign of the twist phase.

**Figure 4:**
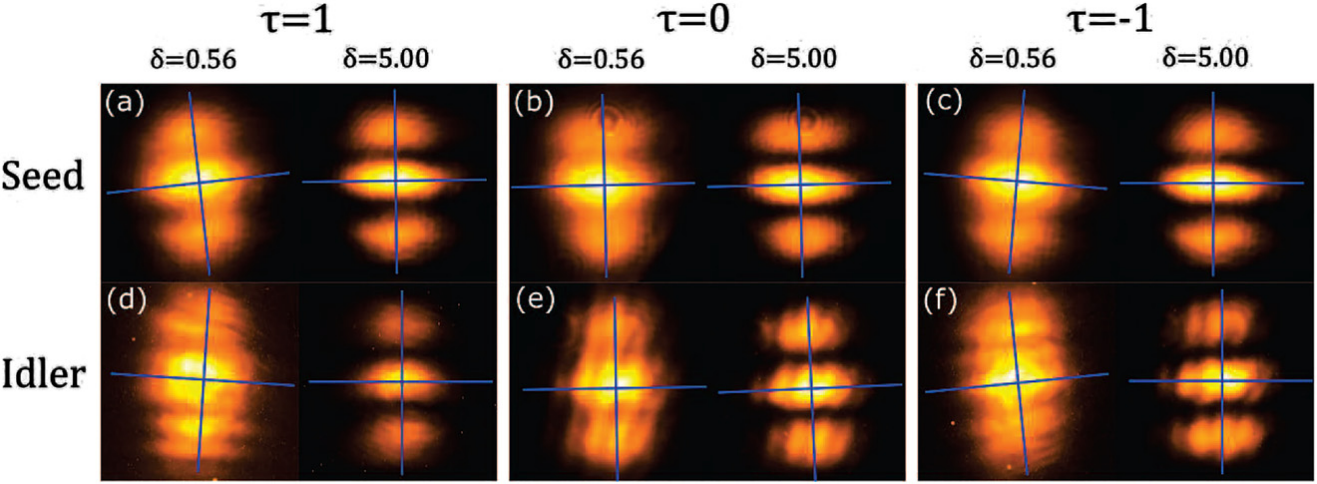
Interference intensity patterns of double-slits compared between two different transverse coherence lengths, *δ* = 0.56 mm (left in each panel) and *δ* = 5.00 mm (right in each panel). The coordinate systems represent the rotation angles given in the text. The seed beam (a) undergoes a counterclockwise rotation with normalized twist phase *τ*_*s*_ = 1, (b) does not rotate when *τ*_*s*_ = 0, and (c) undergoes a clockwise rotation with *τ*_*s*_ = −1. The idler beam, as the phase conjugate of the seed, rotates in the opposite direction. We see in (d) a clockwise rotation when *τ*_*s*_ = 1, (e) close to zero rotation for *τ*_*s*_ = 0, and in (f) a counterclockwise rotation when *τ*_*s*_ = −1.

Figure 4 shows interference patterns for both seed and idler fields for normalized twist phase of the seed beam *τ*_*s*_ = 0, ±1, and two values of the transverse coherence. For a qualitative demonstration we choose two coherence length values that, combined between visibility and rotation angle, best illustrate the rotation. For *τ*_*s*_ = 1, we obtained the results presented in Figure 4a and d for the seed and idler beams, respectively. In these cases, as in Figure 4c and f with *τ*_*s*_ = −1, for *δ*_*s*_ = 0.56 mm, we can observe the seed and idler beams rotate in opposite directions. For *τ*_*s*_ = 0, as in Figure 4b and e, we observe very little rotation of the images, as expected. We include the results for *δ*_*s*_ = 5.0 mm, which show only very small tilt, to further demonstrate that observed rotation is a result of the twist phase (*μ* ∝ 1/*δ* ^2^), and not other aspects of the beam.

To quantify the rotations, we use a Radon transform of the images to identify the rotation angle as the one which maximizes the marginal visibility of the interference pattern. We obtained data given in Table 1, where we calculate *μ* = *θ*_*μ*_/*f* after converting *θ*_*μ*_ to radians, for both seed and idler. Also given is the theoretical value of the twist phase for the seed beam. We can see that the numerical values obtained are consistent with the TGSM idler beam being the phase conjugate of the TGSM seed beam.

**Table 1:** Twist phase of signal and idler beams obtained from the interference patterns shown in Figure 4.

Theory (×10^3^ mm^−1^)	*μ*_*s*_ = 0.40	*μ*_*s*_ = 0	*μ*_*s*_ = −0.40
*μ*_*s*_ (×10^3^ mm^−1^)	0.26 ± 0.04	−0.02 ± 0.04	−0.13 ± 0.04
*μ*_*i*_ (×10^3^ mm^−1^)	−0.15 ± 0.04	−0.04 ± 0.04	0.22 ± 0.04
**Theory (×10^3^ mm^−1^)**	*****μ***_***s***_ **=** **0.005****	***μ***_***s***_ **=** **0**	***μ***_***s***_ **=** **−0.005**
*μ*_*s*_ (×10^3^ mm^−1^)	0.04 ± 0.04	0.00 ± 0.04	0.04 ± 0.04
*μ*_*i*_ (×10^3^ mm^−1^)	0.02 ± 0.04	0.02 ± 0.04	−0.02 ± 0.04

The top (bottom) three rows correspond to coherence length *δ*_*s*_ = 0.56 mm (*δ*_*s*_ = 5.0 mm). The expected twist phase for *δ*_*s*_ = 5.0 mm is smaller than our experimental precision. The idler beam takes on a twist phase that is consistent with the conjugation of the seed twist phase.

## 5 Conclusions

We have studied StimPDC in the case where the pump and seed beams have partial transverse coherence. In particular, when the pump and seed beams are described by the TGSM, we have shown analytically that the stimulated idler beam is also a TGSM beam, with properties determined by those of the pump and seed. These results were investigated experimentally using a pump beam with large transverse coherence. In this case, we experimentally confirm our theoretical results, showing that the coherence properties of the idler are determined by the seed beam alone. We have also demonstrated that the TGSM idler beam is the phase conjugate of the TGSM seed beam. These results open up the possibility of using stimulated down-conversion in schemes for correction of distortions in the wavefront of partially coherent beams. Our results motivate the use of the GSM and TGSM fields in communication protocols or imaging scenarios where light beams propagate through disturbing media. An interesting avenue for future research concerns polarization-induced spatial incoherence, and/or partial polarization, which can be addressed using a two-crystal source as in Refs. [34, 36]. We note that the control of the degree of polarization might allow for the generation of TGSM-like beams without the use of movie-masks in an SLM.
